# (−)-*Epi*afzelechin Protects against Ovariectomy-induced Bone Loss in Adult Mice and Modulate Osteoblastic and Osteoclastic Functions In Vitro

**DOI:** 10.3390/nu9050530

**Published:** 2017-05-22

**Authors:** Ka-Chun Wong, Sisi Cao, Xiaoli Dong, Man-Chun Law, Tak-Hang Chan, Man-Sau Wong

**Affiliations:** 1 State Key Laboratory of Chinese Medicine and Molecular Pharmacology (Incubation), Shenzhen 518057, China; aguesses@yahoo.com.hk (K.-C.W.); sissi.cao@connect.polyu.hk (S.C.); 2 Department of Applied Biology and Chemical Technology, The Hong Kong Polytechnic University, Hung Hom, Kowloon, Hong Kong, China; xiaoli.dong@polyu.edu.hk (X.D.); manchunlaw@hotmail.com (M.-C.L.); tak-hang.chan@polyu.edu.hk (T.-H.C.); 3 Shenzhen Key Laboratory of Food Biological Safety Control, Shenzhen 518057, China; 4 Department of Chemistry, McGill University, Montréal, QC H3A 0B8, Canada

**Keywords:** (−)-*epi*afzelechin, flavan-3-ol, ovariectomised, osteogenesis, osteoclastogenesis, trabecular bone

## Abstract

The present study was designed to characterize the bone protective effects of (−)-*epi*afzelechin (EAF), a flavan-3-ol, in mature ovariectomized mice model and its ability to stimulate osteoblastic activity and inhibit osteoclastic activity. Mature C57BL/6 mice (three to four months old) were either ovariectomised (OVX) or sham-operated and subjected to treatment (vehicle, 17β-oestradiol (E2, 200 μg/kg/day) or EAF (500 μg/kg/day) orally for six weeks. EAF and E2 significantly reduced urinary calcium (Ca) excretion, serum osteocalcin (OCN), and urinary deoxy-pyridinoline (DPD); increased bone mineral density (BMD); and improved micro-architectural properties in OVX mice. EAF significantly increased cell viability, alkaline phosphatise (ALP) activity, and collagen content, as well as runt-related transcriptional factor 2 (Runx2) mRNA expression in murine osteoblastic MC3T3-E1 cells. In addition, EAF significantly reduced the viability of osteoclast precursor murine leukemia monocyte RAW 264.7 cells and tartrate-resistant acid phosphatase (TRAP) activities in mature osteoclastic RAW 264.7 cells. EAF is a bioactive flavan-3-ol that protects estrogen deficiency-induced bone loss in OVX mice and exerts direct modulating effects in bone cells in vitro.

## 1. Introduction

Recent epidemiological studies have reported the association between flavonoid (the most common group of plant polyphenols) intakes and markers of bone health [1,2,3,4] in different female populations, including Scottish peri-menopausal women [1], TwinsUK Cohort (population of women aged 18–79 years) [2], postmenopausal Chinese women (aged 56–63 years) [3], and elderly Australian women (aged ≥ 75 years) [4]. Total flavonoid intakes were found to positively associate with the bone mineral density (BMD) at the femoral neck and lumbar spine in the Scottish population [1] and postmenopausal Chinese women [3] as well as the BMD at the spine of the TwinsUK cohort [2]. Subclasses analysis of the contribution of different flavonoid intakes indicated that catechins (a sub-group of the flavan-3-ols) and flavanones were negatively associated with bone resorption markers in the Scottish population [1]; flavan-3-ol from tea was positively associated with BMD in postmenopausal Chinese women [3], while anthocyanins were most strongly associated with BMD in the hips and spines of the TwinsUK cohort [2]. Most importantly, a recent study by Myer et al. [4] reported that higher intake of black tea and particular classes of flavonoids were associated with lower risk of fracture-related hospitalizations in the elderly women at high risk of fracture in Australia. These studies support the role of flavonoids present in plant-based foods on bone health.

**Figure 1:**
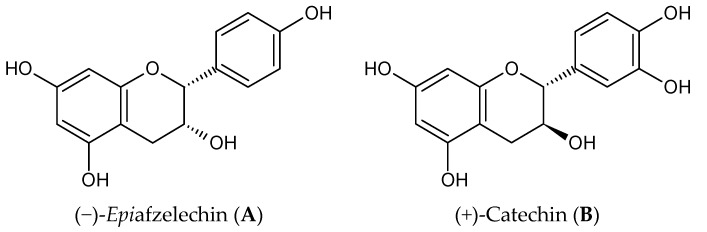
Chemical structure of (−)-*epi*afzelechin (**A**) and (+)-catechin (**B**).

Different subclasses of flavonoids have been shown to be effective in protecting against ovariectomy-induced bone loss in preclinical studies [5,6,7,8], which include isoflavones (e.g., genistein from soybeans) [9], flavanones (glycosides) (e.g., naringin from grapefruit) [5], flavonols (glycosides) (e.g., icariin from *Herba Epimedii*) [8], and flavanols (e.g., *epi*galloacatechin-3-gallate (EGCG) from tea) [7]. Soy isoflavones had been shown to stimulate osteoblastic functions [10] and suppress the production of osteoclastogenesis-regulatory cytokines [11]. Their actions in bone are believed to be mediated by binding to estrogen receptor (ER) α or β, which subsequently leads to the alteration of gene transcription via interaction with estrogen response elements (EREs) in the promoters of target genes [12]. However, concerns have been raised regarding the potential unintended side effects of soy isoflavones in other estrogen sensitive tissues [13]. On the other hand, the most commonly consumed flavonoids in both Western and Asian countries were flavan-3-ols, especially those found in tea. In vitro studies suggest that epigallocatechin gallate (EGCG), the most abundant catechin flavan-3-ol in green tea extract, might exert bone protective effects via its anti-oxidative and anti-inflammatory actions as well as its abilities to promote osteoblastogenesis [14,15,16] and inhibit osteoclastogenesis [17]. However, it should be noted that EGCG is easily oxidized when dissolved in water, and its bioavailability is relatively low, possibly due to its short half-lifein vivo [18]. The effective concentrations of tea catechins required for osteoprotective effects in most studies (1–100 μM) are well above the normal physiological concentrations in tea drinkers [19]. There is therefore a need to find new flavonoids that can offer effective osteoprotective abilities at lower concentrations with fewer undesirable side effects. 

(−)-*Epi*afzelechin (1, EAF, Figure 1) is found in plants such as *Celastrus orbiculatus* [20], *Cassia sieberiana* [21], *Typha capensis*, *Drynariae fortune* [22], and *Camellia sinensis* [23]. The leaves and leave buds of *Camellia sinensis* are used for the preparation of tea. In a bioactivity-guided fractionation of Huangshan Maofeng tea, EAF was isolated together with other catechins. In vitro studies indicated that EAF could stimulate cell proliferation and differentiation of rat osteoblasts and significantly increase the area of mineralized bone nodules. However, it is unclear if EAF could exert bone protective effects in vivo and its effects on the process of bone formation and bone resorption have not been systematically evaluated. Recently, we have developed a method for the total chemical synthesis of EAF [24] as well as a bioanalytical method for detecting its levels in mice plasma to determine its bioavailability in vivo [25]. These latest developments enable us to have sufficient material for the evaluation of its in vivo efficacy.

The present study aimed toevaluate the bone protective effects of EAF in vivoand to characterize the effects of EAF on bone formation and bone resorption in vitro.It is our hope that the study will provide evidence for its use as a drug candidate for the prevention and management of postmenopausal osteoporosis.

## 2. Materials and Methods

### 2.1. Chemicals

(3-(4,5-Dimethylthiazol-2-yl)-5-(3-carboxymethoxyphenyl)-2-(4-sulfophenyl)-2H-tetrazolium) MTS waspurchased from Promega (Promega, Madison, WI, USA). 17β-Oestradiol (E2), acetic acid, pepsin, and ascorbic acid were obtained from Sigma Aldrich (Sigma, St. Louis, MO, USA). β-Glycerophoshpate (β-GP) was obtained from Calbiochem (Calbiochem, San Diego, CA, USA), and ICI 182780 was obtained from Tocris (Tocris, Bristol, UK). (−)-*Epi*afzelechin (EAF) was synthesized as previously described [26] and characterized by high-performance liquid chromatography (HPLC), electrospray ionisation mass spectrometry (ESI-MS), and nuclear magnetic resonance spectroscopy (NMR). The purity of synthetic EAF was >99%.

### 2.2. Animal Experiments

Three to four-month-old mature C57BL/6J mice were purchased from the Laboratory Animal Services Centre (The Chinese University of Hong Kong, HK, China). All mice were housed in a room at 22 °C, provided with a 12-h light and dark cycle. Mice (*n* = 34) subjected to either sham-operated or ovariectomised (OVX) operations were orally administrated for six weeks as follows: Sham + vehicle (Sham, *n* = 9), OVX + vehicle (OVX, *n* = 9), OVX + 17β-oestradiol (E2, 200 μg/kg/day, *n* = 8), or OVX + EAF (EAF, 500 μg/kg/day, *n* = 8). All mice were pair-fed with approximately 3 g/day, the minimum daily average food intake, of phytoestrogen-free AIN-93M rodent diet (Research diets, New Brunswick, NJ, USA). One day before sacrifice, mice were individually housed in a metabolic cage for urine collection. The mice were then sacrificed by cardiac stick exsanguinations under anesthesia. Serum was prepared in aliquots and stored at −80 °C for biochemical measurements. The wet weight of the uterus was measured for the uterine index. The left tibia and intact lumbar vertebra were collected and stored at −20 °C for micro-computed tomography (μCT) analysis. The experimental protocol was conducted under the animal license issued by the Department of Health, the Hong Kong SAR Government, and the Animal Subjects Ethics Sub-committee (ASESC No. 12/41) of the Hong Kong Polytechnic University.

### 2.3. Biochemical Assays of Serum and Urine Samples

The calcium and inorganic phosphorus concentrations in both serum and urine samples were determined by standard colorimetric methods using commercial kits (STANBIO laboratory, Boerne, TX, USA). The serum osteocalcin (OCN) level was measured by a mouse osteocalcin ELISA kit (Alfa Aesar, Lancashire, UK). The urinary deoxypyridinoline (DPD) level was measured by using a DPD enzyme immunoassay kit (METRA™ Quidel Corporation, San Diego, CA, USA). The urinary calcium (Ca), phosphorus (P), and DPD levels were normalized with urinary creatinine (Cr) concentration.

### 2.4. Microcomputed Tomography (μCT)

The left tibia and fourth lumbar vertebra (L4) were scanned at high resolution (10.5 μm) by a microcomputed tomography (μCT) system (viva-CT40; Scanco Medical, Bassersdorf, Switzerland) using an integration time of 30 ms, energy of 70 kVp, and intensity of 114 mA. For the left proximal tibia, 100 μCT slices were acquired from the metaphyseal growth plate, in which the volume of interest was contoured from 50 serial slices starting from the disappearance of the condyle, corresponding to a 0.525 mm region, for evaluation. A semi-automatic contouring method was applied to segment the trabecular bone compartment. Similarly, for lumbar vertebra (L4), 150 μCT slices were acquired distal from the growth plate. The volume of interest was contoured from 100 slices to generate three-dimensional images for all samples. A constant threshold of 300 was used to evaluate bone properties.

### 2.5. Culture of Murine Pre-osteoblastic MC3T3-E1

Murine pre-osteoblastic MC3T3-E1 (subclone 14) cells (ATCC, CRL-2594, Manassas, VA, USA) were cultured in modified Eagle medium alpha (MEMα, Gibco, Carlsbad, CA, USA), supplemented with 10% fetal bovine serum (FBS, Gibco, Carlsbad, CA, USA), 100 U/mL penicillin, and 100 µg/mL streptomycin (Gibco, Carlsbad, CA, USA). MC3T3-E1 differentiation medium, consisting of phenol-red free MEMα with 10% FBS, 100 U/mL penicillin, 100 µg/mL streptomycin, 25 μg/mL of ascorbic acid, and 10 mM of β-glycerophosphate (GP), was used to induce the differentiation of MC3T3-E1 during EAF treatment. For cell proliferation assay, MC3T3-E1 cells were treated with phenol-red free routine culture medium with 10^−8^ M of E2, 10^−10^ to 10^−6^ M of EAF, or its vehicle for 24 h. The cell viability was determined by MTS assay. For the cell differentiation study, MC3T3-E1 cells in differentiation medium were treated with 10^−8^ M of E2, 10^−10^ to 10^−6^ M of EAF, or its vehicle for seven days. Cells were lysed with passive lysis buffer (Promega, Madison, WI, USA) and collected for alkaline phosphatase activity determination using a Wako lab assay ALP (Wako Pure Chemical Industries, Osaka, Japan). The protein content of cell lysate was determined by the Bradford protein assay (Bio-Rad, Philadelphia, PA, USA). For extracellular matrix collagen content, the acid-pepsin soluble collagen was extracted from cell layers by 0.5 M acetic acid and 0.1 mg/mL pepsin at 4 °C for 16 h. The lysate was collected for collagen analysis using the soluble collagen assay (Sircol, Carrickfergus, County Antrim, UK). For mineralization, MC3T3-E1 cells were treated with differentiation medium with 10^−8^ M of E2, 10^−10^ to 10^−6^ M of EAF, or its vehicle for 21 days. The cells were washed, fixed, and stained with 1% Alizarin Red S (Sigma). The stained cell images were captured for visual comparison. The stained cultures were further dissolved in 0.5 M HCl and 5% sodium dodecyl sulfate (SDS) and subjected to optical density measurement at 415 nm by a spectrophotometric plate reader (Bio-Rad, Philadelphia, PA, USA) for an assessment of the degree of mineralization.

### 2.6. Gene Expression of MC3T3-E1 Cells

Total RNA was extracted from treated cells using Trizol reagents (Invitogen, Carlsbad, CA, USA), as described in the manufacturer’s protocol, and reverse transcripted to construct the complementary cDNA by a cDNA synthesis kit (Applied Biosystems, Carlsbad, CA, USA). cDNA was then diluted for real time polymerase chain reaction (PCR) analysis by using 7900HT (Applied Biosystems) and EvaGreen real time PCR supermix (Bio-rad). The following primers were used: runt-related transcriptional factor 2 (Runx2, 57 °C): forward-gTCAgCAAAgCTTCTTTTgg and reverse-TTgTTgCTgTTgCTgTTgTT; Collagen (Col-1α1, 58 °C): forward-AATggTgCTCCTggTATTgC and reverse-ggCACCAgTgTCTCCTTTgT; and β-actin (56 °C): forward-AAgAgCTATgAgCTgCCTgA and reverse-TggCATAgAggTCTTTACgg. The concentrations of unknown samples were calculated by fitting the Ct value to the standard curve by the SDS software package (Applied Biosystems).

### 2.7. Culture of Murine Leukemia RAW 264.7 Cells

Murine leukemia monocyte RAW 264.7 cells (ATCC, TIB-71, Manassas, VA, USA) were cultured in Dulbecco’s Modified Eagle’s Medium (DMEM, ATCC), supplemented with 10% FBS (Gibco, Carlsbad, CA, USA), 100 U/mL penicillin, and 100 µg/mL streptomycin (Gibco, Carlsbad, CA, USA). Osteoclast differentiation medium, composed of phenol-red free MEMα with 10% FBS, 100 U/mL penicillin, 100 µg/mL streptomycin, and 100 ng/mL receptor activator of NF-κB ligand (RANKL, Millipore GF091, Billerica, MA, USA), were used to induce the differentiation of RAW 264.7 cells during EAF treatment. For cell proliferation studies, RAW 264.7 cells were treated with phenol-red free MEMα medium with 10^−8^ M E2, vehicle, and 10^−10^ to 10^−6^ M of EAF for 24 h. The cell viability was determined by MTS assay as described above. To determine the effects on differentiation, RAW 264.7 cells were induced by differentiation medium containing RANKL and treated with 10^−8^ M of E2, 10^−10^ to 10^−6^ M of EAF, or its vehicle for five days. Treated cells were stained for Tartrate Resistant Acid Phosphatase (TRAP) expression by using an acid phosphatase staining kit (Sigma). TRAP-positive multinucleated cells showing more than three nuclei were counted as mature osteoclasts. The TRAP activities of RAW 264.7-derived osteoclasts were measured by the acid phosphatase assay kit (BioVision, Milpitas, CA, USA).

### 2.8. Statistical Analysis

The results are reported as mean ± standard error mean (SEM). For both in vivo and in vitro studies, statistical differences between group means were evaluated by one-way ANOVA analysis (Prism 6.0 for Windows, GraphPad, CA, USA), followed by using the Tukey’s post-test. A *p* value less than 0.05 is considered statistically significant.

## 3. Results

### 3.1. EAF Suppressed Body Weight Gain, Excessive Urinary Ca, and Bone Markers in OVX Mice

As shown in Table 1, body weight was significantly increased in OVX mice upon treatment for six weeks (vs. Sham). Treatment of OVX mice with estrogen (E2) and EAF significantly reduced the OVX-induced increase in body weight (vs. OVX). As expected, estrogen deficiency induced by ovariectomy resulted in the atrophy of the uterus, as reflected by the significant decrease of the uterus index in OVX mice (vs. Sham, Table 1). The treatment of OVX mice with E2, but not EAF, significantly increased the uterine index (vs. OVX). Ovariectomy significantly increased urinary Ca excretion in mice (vs. Sham). Treatment with E2 and EAF significantly reduced urinary Ca excretion in OVX mice (vs. OVX). Serum Ca, serum P, and urinary P excretion were not significantly different amongst treatment groups. Ovariectomy significantly increased serum osteocalcin (OCN) levels and urinary deoxypyridinoline (DPD) levels in mice (vs. Sham), indicating an increase in bone turnover rate in OVX mice. Treatment with EAF and E2 significantly suppressed OVX-induced serum OCN levels and urinary DPD levels in OVX mice (vs. OVX). 

### 3.2. EAF Improved Bone Properties of Proximal Tibia and Lumbar Vertebra (L4) in OVX Mice

**Figure 2:**
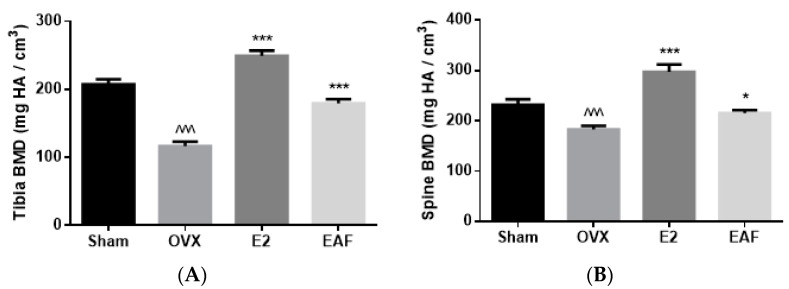
(−)-*epi*afzelechin (EAF) improved bone mineral density (BMD) in the proximal tibia (**A**) and lumbar vertebra L4 (**B**) of ovariectomized C57BL/6J mice. Mature ovariectomised (OVX) or sham-operated (Sham) C57BL/6J mice (three to four months old) paired-fed with phytoestrogen-free AIN-93M diet were treated with vehicle (Sham or OVX), E2 (200 μg/kg/day) or EAF (500 μg/kg/day) for six weeks. The left tibia and lumbar vertebra were collected upon sacrifice (*n* = 8–9 for each group). BMD was determined using μCT scanning as described in Methods. Data werepresented as mean ± SEM and analyzed by one-way ANOVA followed by Tukey’s multiple comparison test. ^^^ *p* < 0.001 vs. Sham; * *p* < 0.05, ** *p* < 0.01, and *** *p* < 0.001 vs. OVX.

The effects of OVX, E2, and EAF on bone mineral density (BMD) and micro-architectural properties at proximal tibia and lumbar vertebra (L4) are shown in Figure 2 and Table 2. Ovariectomy significantly reduced trabecular BMD at the proximal tibia and L4 in mice by 43% and 21%, respectively (vs. Sham, Figure 2). Treatment with E2 significantly increased trabecular BMD at the proximal tibia by 114% and at L4 by 62% in OVX mice (vs. OVX, Figure 2). Similarly, treatment with EAF significantly increased trabecular BMD at the proximal tibia by 53% and at L4 by 17.5% in OVX mice (vs. OVX, Figure 2). In addition, the bone microarchitecture at proximal tibia and L4 in OVX mice were weakened in response to ovariectomy, as revealed by the reduction of bone volume/total volume (BV/TV), trabecular number (Tb.N), trabecular thickness (Tb.Th), connectivity density (Conn.D) and the induction of trabecular separation (Tb.Sp) and structural model index (SMI) (vs. Sham, Table 2). Treatment with E2 and EAF significantly improved bone properties at both sites, as revealed by increasing BV/TV, Tb.N, Tb.Th, and Conn.D and decreasing Tb.Sp and SMI at both sites in OVX mice (vs. OVX, Table 2).

### 3.3. EAF Induced Osteoblastic Function in MC3T3-E1 Cells

**Figure 3:**
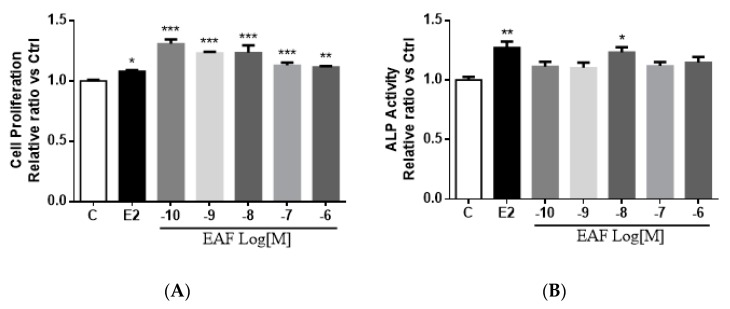

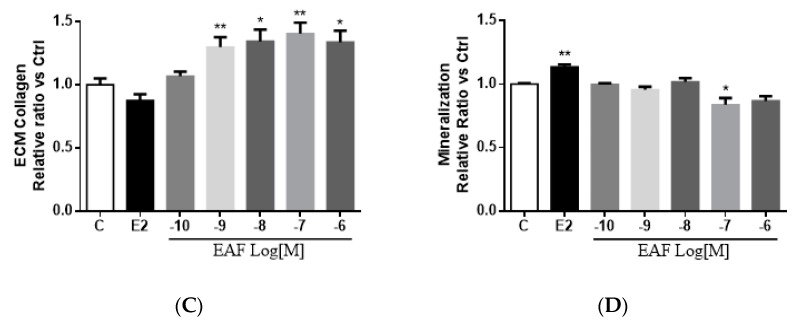
(−)-*epi*afzelechin (EAF) stimulatedosteoblastic functions in MC3T3-E1 cells. (**A**) Cell proliferation was determined by MTS assay upon treatment of MC3T3-E1 cells with 10^−8^ M of E2, 10^−10^ to 10^−6^ M of EAF, or its vehicle (1% EtOH *v/v*) for 1day; (**B**) Alkaline phosphatase activities and (**C**) collagen content were determined upon treatment of cells with 10^−8^ M of E2, 10^−10^ to 10^−6^ M of EAF, or its vehicle (1% EtOH *v/v*) for seven days; (**D**) Mineralization was determined by Alizard Red S staining upon treatment of cells with 10^−8^ M of E2, 10^−10^ to 10^−6^ M of EAF, or its vehicle (1% EtOH *v/v*) for 21 days. The stained plates were dissolved in 5% SDS and 0.5 M HCl for colorimetric determination. The relative cell proliferation rate, alkaline phosphatase (ALP) activity, collagen content, and degree of mineralization were presentedas mean ± SEM value with *n* = 3. * *p* < 0.05, ** *p* < 0.01, and *** *p* < 0.001 vs. vehicle.

As shown in Figure 3A, EAF and E2 significantly increased cell proliferation in MC3T3-E1 cells (vs. vehicle). EAF at 10^−10^ M appeared to be the most effective dose and significantly increased the cell proliferation rate by 31% in MC3T3-E1 cells. MC3T3-E1 cells were pre-treated with differentiation media containing ascorbic acid and inorganic phosphate [27,28] to induce its differentiation into mature osteoblasts. Both EAF and E2 at 10^−8^ M significantly increased alkaline phosphatase (ALP) activities in pre-treated MC3T3-E1, suggesting that EAF could enhance osteoblastic differentiation (Figure 3B). EAF at 10^−10^ to 10^−6^ M, but not E2, significantly increased extracellular matrix (ECM) collagen levels in pre-treated MC3T3-E1 cells (Figure 3C). EAF at 10^−8^ M significantly induced ALP activities and collagen levels in pre-treated MC3T3-E1 cells by 23% and 34%, respectively. To determine if EAF could induce osteoblast maturation, the ability of MC3T3-E1 cells to form mineralized nodules was determined. As shown in Figure 3D, the mineralization of MC3T3-E1 was significantly increased upon treatment with E2 but not with EAF. Indeed, at a concentration of 10^−7^ M or higher, EAF appeared to significantly reduce the formation of mineralization nodules in MC3T3-E1 cells. The results indicated that EAF induced osteoblast differentiation but not maturation, suggesting that the actions of EAF on bone formation occur at the early stage of osteoblastogenesis.

### 3.4. EAF Increased Osteoblast-specific mRNA Expression in MC3T3-E1 Cells

**Figure 4:**
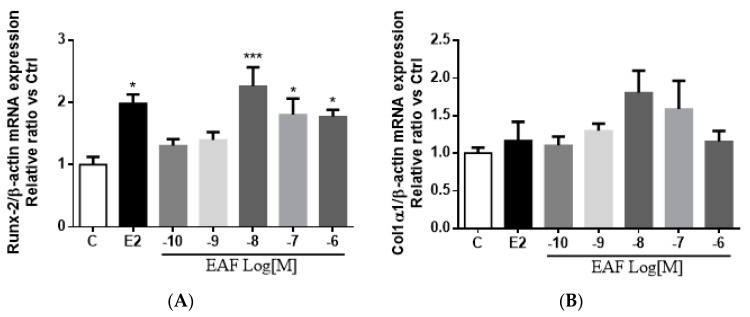
EAF stimulatedosteoblast-specific mRNA expression in MC3T3-E1 cells. The mRNA expression level of (**A**) Runx2 and (**B**) Col 1α1 in MC3T3-E1 cells upon treatment with 10^−8^ M of E2, 10^−10^ to 10^−6^ M of EAF, or its vehicle (1% EtOH *v/v*) for three days was determined by reverse-transcriptase real time polymerase chain reaction analysis. The relative gene expressions were expressed as mean ± SEM value. * *p* < 0.05, and *** *p* < 0.001 vs. control.

To determine if EAF regulates osteoblastic differentiation at the transcriptional level, the mRNA expression of an osteoblast-specific transcriptional factor, Runt related transcription factor 2 (Runx2) [27,28], as well as collagen 1α1 in MC3T3-E1 cells, was determined. Treatment with E2 at 10^−8^ M or EAF at 10^−8^ M to 10^−6^ M for three days significantly increased the Runx2 mRNA expression by 130% and 77% in pre-treated MC3T3-E1 cells, respectively (Figure 4A). Moreover, EAF, but not E2, appeared to induce collagen 1α1 mRNA expression at 10^−8^ M in pre-treated MC3T3-E1 cells, but the change was not statistically significant (Figure 4B). This result was in line with the results of ECM collagen assay as EAF, but not E2, could significantly increase the collagen content in pre-treated MC3T3-E1 cells.

### 3.5. EAF Reduced Osteoclastic Activities in Receptor Activator of NF-κB Ligand (RANKL) Induced RAW 264.7 Cells

**Figure 5:**
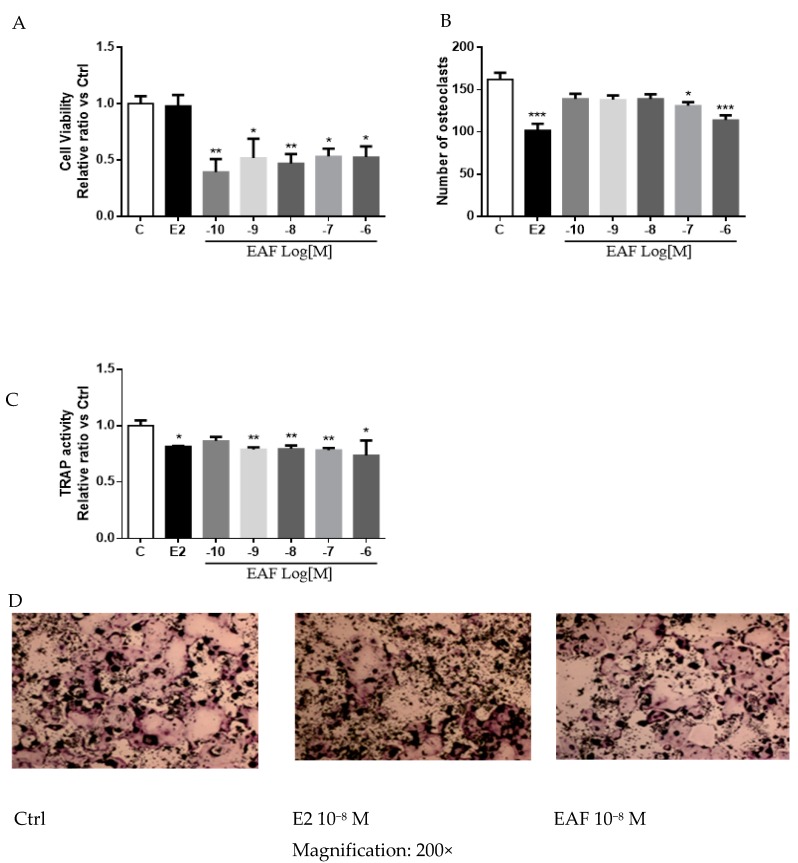
EAF decreased cell viability (**A**), reduced number of tartrate resistant acid phosphatase (TRAP) stained multinucleated cells (**B**) and TRAP activities (**C**) in Receptor Activator of NF-κB Ligand (RANKL)-induced RAW264.7 cells. (**D**) The representative TRAP staining of RANKL-induced RAW 264.7 cells.

To determine if EAF exerts direct effects on osteoclastic functions, RAW 264.7 cells, used as the precursor cells of osteoclasts [29], were treated with 10^−10^ to 10^−6^ M EAF, 10^−8^ M E2, or vehicle for 24 h. As shown in Figure 5A, EAF at 10^−10^ to 10^−6^ M, but not E2, significantly reduced the growth of un-induced RAW 264.7 cells. The effects of EAF on the process of osteoclast formation were also determined in RAW 264.7 cells induced with RANKL in which tartrate resistant acid phosphatase (TRAP) enzyme activities were used as a marker to evaluate the maturation rate of RAW 264.7 cells [30]. E2 at 10^−8^ M and EAF at 10^−7^ to 10^−6^ M significantly reduced the number of multinucleated cells (Figure 5B) and TRAP-activities (Figure 5C) in RANKL-induced RAW 264.7 cells. Representative TRAP staining of RANKL induced RAW 264.7 cells was shown in Figure 5D. These results indicated EAF could exert direct effects on reducing the maturation rate of RANKL-induced RAW 264.7 cells.

## 4. Discussion

The present study clearly demonstrated that oral administration of EAF, a flavan-3-ol, could protect against ovariectomy-induced bone loss in mice without inducing uterotrophic responses. EAF could suppress the increase in weight gain, urinary Ca excretion, and bone turnover markers (serum osteocalcin and urinary DPD levels) induced by OVX in mice in a way similar to the actions of E2. In addition, micro-CT analysis indicated that EAF could increase the BMD of trabecular bone at both the proximal tibia and lumbar spine in OVX mice. Furthermore, EAF improved bone microarchitecture in OVX mice by increasing BV/TV, Tb.Th, and Conn.D and decreasing Tb.Sp and SMI of the proximal tibia, as well as by increasing BV/TV, Tb.N, and Conn.D and decreasing Tb.Sp and SMI of the lumbar spine. Thus, our results indicated that EAF was effective in protecting against OVX-induced bone loss in mice in a way similar to E2. However, unlike E2, EAF did not increase the uterus index in OVX mice, suggesting that EAF might exert tissue selective estrogen-like effects in vivo.

Our in vitro study using pre-osteoblastic MC3T3-E1 cells showed that EAF exerted anabolic effects in both immature and mature osteoblastic cells. Immature MC3T3-E1 cells proliferate and synthesize DNA rapidly, while mature cells express unique osteoblastic functional markers [31]. Treatment of MC3T3-E1 cells with EAF induced ALP activities and collagen content at most of the tested concentrations, suggesting that EAF induced the maturation of MC3T3-E1 cells by inducing the expression of osteoblastic functional markers. In addition, EAF induced the expression of osteoblast-specific transcriptional factor Runx2 in MC3T3-E1 cells at a concentration of 10^−8^ to 10^−6^ M, suggesting that EAF enhanced the process of osteoblastogenesis. Indeed, Runx2, also recognized as core-binding factor subunit alpha-1 (Cbfa1), is known to play a central role in regulating osteoblastic differentiation and bone formation [32]. Runx2 could bind to osteoblast-specific element 2 [33] and activates bone gamma-carboxyglutamic acid-containing protein (BGLAP, or osteocalcin), collagen 1α1, bone sialoprotein, and osteopontin genes in osteoblasts [34]. However, it should be noted that EAF did not affect the mineralization of MC3T3-E1 cells. These results suggest that EAF is a bone protective phytochemical, and it directly acts on inducing osteoblast formation. 

Our study also demonstrated that EAF could reduce osteoclastic activities in vitro. EAF reduced the viability of osteoclastic precursor cells and the activities of tartrate resistant acid phosphatase (TRAP) in RAW 264.7 cells. Osteoclasts, which express high TRAP activities [30], can be formed in cell cultures from RAW264.7 cells upon induction by RANKL. The measurement of TRAP activity is commonly used for assessing of the degree of maturation of RAW 264.7 cells. Our results demonstrated that the actions of EAF were similar to those of E2 in inhibiting TRAP activities in mature osteoclasts derived from RAW 264.7 cells, indicating that EAF might suppress the formation of mature osteoclasts and the process of bone resorption. However, it should be noted that only EAF, but not E2, could reduce the uninduced RAW 264.7 cells, suggesting that the actions of E2 and EAF are not the same. 

The osteoprotective effects of EAF are found to be more potent than those of genistein, the most common soy phytoestrogen, as well as EGCG and (+)-catechin from tea, which share the same flavan-3-ol structure as EAF. The present study showed that oral administration of EAF at 500 μg/kg/day for six weeks could effectively improve BMD and microarchitecture at the proximal tibia and lumbar spine in mature OVX mice, whereas the effective dose was reported to be 50 mg/kg/day of genistein for four weeks in OVX mice in our previous study [35]. EGCG were reported to prevent bone loss in three month [36] and six month [7] old OVX rats when administrated intra-peritoneally for 12 weeks at 10 mg/kg/day and 3.4 mg/kg/day, respectively. The dosages of EGCG required for protection against ovariectomy-induced bone loss in rats were much higher than the oral dose of EAF being used in the present study. In addition, the effective concentrations of tea catechins in most in vitro studies range from 10 μM to 100 μM, well above the normal physiological concentrations in tea drinkers [19]. In contrast, the effective concentrations of EAF found in our study are ranged from 0.1 nM to 1 μM, with the maximial effect at 10 nM in MC3T3-E1 cells. In rat osteoblastic-like UMR-106 cells, EAF significantly induced its proliferation, even at the concentration of 10^−14^ M, and increased the ALP activity at the concentration of 10^−12^ (unpublished data). The relatively low effective concentration of EAF suggests that it might be superior than tea catechins in exerting osteoprotective effects in vivo, as its circulating level could be more readily achieved through oral consumption. 

The actions of EAF at a lower effective dose on bone formation and bone resorption were found to be similar to those reported for EGCG. EGCG has been shown to promote bone formation via the stimulatory effects on the differentiation of bone marrow mesenchymal stem cells [14], human osteoblastic-like SaOS-2 [15], and MG-63 cells [37] and to suppress bone resorption via the inhibitory effects on osteoclast formation and differentiation [17]. However, a study using murine osteoblastic MC3T3-E1 cells showed that EGCG inhibited ALP activity and did not alter the mineralization of differentiated MC3T3-E1 cells [38], suggesting that the actions might be cell type specific. Future work will be needed to study the effect of EAF in other cell types within a narrow range of dosage to investigate if EAF behaves similarly to EGCG. It should also be noted that EGCG is a weak agonist for ERα (IC_50_: 480 μM vs. 5.7 nM for E2) and ERβ (IC_50_: 97 μM vs. 8.2 nM for E2), while EAF failed to bind to neither ERα nor ERβ (unpublished data). Thus, the observed effects of EAF and EGCG on bone cells might not involve ligand-dependent actions of ERs. Indeed, recent studies indicated that ERs can also be activated via extra-nuclear ER signalling pathways [39], and as such the pathway was shown to mediate the anti-apoptotic actions of estrogens in osteoblasts [40,41]. Thus, it is possible that EAF could exert estrogenic effects in bone cells by ligand-independent activation of ERα via extra-nuclear signalling events. Future study is needed to determine if EAF could activate rapid ERα signaling in osteoblastic cells.

The present study clearly demonstrated that EAF, a naturally occurring flavan-3-ol, could exert bone protective effects in vivo and in vitro. Our results indicated that the effective dosages for achieving bone protective effects of EAF in vivo and in vitro are lower than those reported for other bone protective flavonoids, i.e., 0.5 mg/kg/day and 0.1 nM to 1 μM, respectively. A summary of the actions of EAF in comparison with EGCG is shown in Table 3. With the availability of the chemically synthesized EAF [25] and its demonstrated in vivo efficacy for bone health in preclinical animal models, future studies are warranted to evaluate the safety associated with its long-term use and its effectiveness for management of postmenopausal osteoporosis in humans.

## Figures and Tables

**Table 1:** Effects of (−)-*epi*afzelechin (EAF) on body weight, uterine index, and biochemical parameters in ovariectomised C57BL/6J mice.

	Body Weight (% of Change)	Uterine Index (mg/g)	Serum Ca (mg/dL)	Serum P (mg/dL)	Urinary Ca/Cr (mg/mg)	Urinary P/Cr (mg/mg)	Serum OCN (ng/mL)	Urinary DPD (nmol/mmol)
Sham	1.25 ± 0.66	0.32 ± 0.03	8.16 ± 0.23	7.57 ± 0.36	0.21 ± 0.03	7.26 ± 0.39	77.2 ± 1.4	9.4 ± 0.5
OVX	7.28 ± 1.29 ^^^	0.09 ± 0.02 ^^^	8.86 ± 0.22	7.60 ± 0.56	0.42 ± 0.04 ^^^	7.55 ± 0.63	86.6 ± 2.9 ^	14.4 ± 1.2 ^^^
E2	−6.59 ± 1.11 ***	0.70 ± 0.08 ***	9.21 ± 0.20	6.72 ± 0.64	0.29 ± 0.02 *	6.46 ± 0.23	72.0 ± 3.1 **	8.2 ± 0.6 ***
EAF	1.43 ± 1.01 **	0.13 ± 0.01	9.01 ± 0.14	7.22 ± 0.28	0.23 ± 0.04 **	6.74 ± 0.42	78.1 ± 2.2 *	10.3 ± 0.7 *

OVX, ovariectomy; Urinary Ca/Cr, urinary Ca to creatinine ratio; urinary P/Cr, urinary P to creatinine ratio; OCN, osteocalcin; DPD, deoxy-pyridinoline. Body weight (% of change) from baseline to six weeks. Data were presented as mean ± SEM and analyzed by one-way ANOVA followed by Tukey’s multiple comparison test. ^ *p* < 0.05, and ^^^ *p* < 0.001 vs. Sham; * *p* < 0.05, ** *p* < 0.01, and *** *p* < 0.001 vs. OVX.

**Table 2:** Effect of EAF on bone mineral density (BMD) and bone microarchitecture at the proximal tibia and lumbar vertebra L4 in ovariectomised (OVX) mice analyzed by micro-CT.

**Tibia**	**BV/TV (%)**	**Tb.N (mm^−1^)**	**Tb.Th (mm)**	**Tb.Sp (mm)**	**Conn.D (mm^3^)**	**SMI**
Sham	23.3 ± 1.1	4.51 ± 0.24	0.051 ± 0.001	0.175 ± 0.011	196.1 ± 15.9	1.52 ± 0.13
OVX	11.5 ± 1.0 ^^^	3.08 ± 0.37	0.043 ± 0.002 ^^^	0.312 ± 0.035 ^^^	105.6 ± 8.3 ^^	2.26 ± 0.12 ^^^
E2	27.9 ± 1.4 ***	5.38 ± 0.29 ***	0.054 ± 0.001 ***	0.126 ± 0.017 ***	237.2 ± 27.7 ***	1.54 ± 0.13 ***
EAF	19.3 ± 0.6 ***	3.71 ± 0.15	0.050 ± 0.001 ***	0.210 ± 0.005 **	159.4 ± 15.3 *	1.85 ± 0.05 *
**Spine**	**BV/TV (%)**	**Tb.N (mm^−1^)**	**Tb.Th (mm)**	**Tb.Sp (mm)**	**Conn.D (mm^3^)**	**SMI**
Sham	29.4 ± 1.1	4.62 ± 0.12	0.063 ± 0.001	0.154 ± 0.006	141.1 ± 7.1	0.88 ± 0.08
OVX	21.1 ± 0.6 ^^^	3.82 ± 0.08 ^^^	0.058 ± 0.002	0.210 ± 0.005 ^^^	100.2 ± 4.9 ^^^	1.37 ± 0.04 ^^^
E2	37.0 ± 1.7 ***	4.89 ± 0.11 ***	0.075 ± 0.002 ***	0.130 ± 0.006 ***	139.7± 4.3 ***	0.63 ± 0.08 ***
EAF	25.2 ± 0.7 **	4.42 ± 0.06 ***	0.057 ± 0.001	0.169 ± 0.003 ***	134.0 ± 1.7 ***	0.82 ± 0.09 ***

Mature ovariectomised (OVX) or sham-operated (Sham) C57BL/6J mice (three to four months old) paired-fed with a phytoestrogen-free AIN-93M diet were treated with vehicle (Sham or OVX), E2 (200 μg/kg/day), or EAF (500 μg/kg/day) for six weeks. The tibia and lumbar vertebra (L4) were collected upon sacrifice (*n* = 8–9 for each group) and scanned at high resolution by micro-CT system (viva-CT40, Scanco Medical, Switzerland). Bone microarchitecture parameters: bone volume/tissue volume (BV/TV), trabecular number (Tb.N), trabecular thickness (Tb.Th), trabecular separation (Tb.Sp), structural model index (SMI), and connectivity density (Conn.D). Data were presented as mean ± SEM and analyzed by one-way ANOVA followed by Tukey’s multiple comparison test. ^^ *p* < 0.01 and ^^^ *p* < 0.001 vs. Sham; * *p* < 0.05, ** *p* < 0.01, and *** *p* < 0.001 vs. OVX.

**Table 3:** Comparison of the actions of EAF and *epi*galloacatechin-3-gallate (EGCG).

	EAF	EGCG
Osteoblast cell line	↑Proliferation (0.1 nM–1 μM), ↑ALP activity (10 nM), ↑mRNA expression of collagen, Runx2 at (10 nM–1 μM)	↑ALP activity, ↑mineralization via ↑mRNA expression of Runx2, osterix, OC, ALP at 1–100 μM [18]
Osteoclast cell line	↓Proliferation and TRAP activity at 1 nM–1 μM	↓Proliferation and TRAP activity at 10–100 μM [18]
Bone protection in vivo	Prevent bone loss in 3-mo-old OVX mice after gavage of 0.5 mg/kg/day EAF for 6 weeks	Prevent bone loss in 12-week old OVX rat after i.p. of 10 mg/kg/day EGCG for 12 weeks [35]
		Prevent bone loss in 6-month-old OVX rat after i.p. of 3.4 mg/kg/day EGCG for 12 weeks [7]
PK study	The maximum concentrations (Cmax) of (−)-*epi*afzelechin in blood by i.v. and i.p. injection of 10 mg/kg to C57BL/6J mice were found to be 10.6 and 6.0 μg/mL [24]	Cmax of EGCG by a single oral dose EGCG (2 mg/kg) to eight human subjects was 77.9 ± 22.2 ng/mL [42]
		Cmax of EGCG by i.v. (10 mg/kg) and i.g. (75 mg/kg) administration of EGCG to rat were 4.7 ± 0.9 μg/mL and 19.8 ± 3.5 ng/mL, respectively [43]

## References

[1] Hardcastle A.C., Aucott L., Reid D.M., Macdonald H.M. (2011). Associations between dietary flavonoid intakes and bone health in a Scottish population. J. Bone Miner. Res..

[2] Welch A., MacGregor A., Jennings A., Fairweather-Tait S., Spector T., Cassidy A. (2012). Habitual flavonoid intakes are positively associated with bone mineral density in women. J. Bone Miner. Res..

[3] Zhang Z.Q., He L.P., Liu Y.H., Liu J., Su Y.X., Chen Y.M. (2014). Association between dietary intake of flavonoid and bone mineral density in middle aged and elderly Chinese women and men. Osteoporos. Int..

[4] Myers G., Prince R.L., Kerr D.A., Devine A., Woodman R.J., Lewis J.R., Hodgson J.M. (2015). Tea and flavonoid intake predict osteoporotic fracture risk in elderly Australian women: A prospective study. Am. J. Clin. Nutr..

[5] Pang W.Y., Wang X.L., Mok S.K., Lai W.P., Chow H.K., Leung P.C., Yao X.S., Wong M.S. (2010). Naringin improves bone properties in ovariectomized mice and exerts oestrogen-like activities in rat osteoblast-like (UMR-106) cells. Br. J. Pharmacol..

[6] Dai R., Ma Y., Sheng Z., Jin Y., Zhang Y., Fang L., Fan H., Liao E. (2008). Effects of genistein on vertebral trabecular bone microstructure, bone mineral density, microcracks, osteocyte density, and bone strength in ovariectomized rats. J. Bone Miner. Metab..

[7] Chen C.H., Kang L., Lin R.W., Fu Y.C., Lin Y.S., Chang J.K., Chen H.T., Chen C.H., Lin S.Y., Wang G.J. (2013). (−)-*Epi*gallocatechin-3-gallate improves bone microarchitecture in ovariectomized rats. Menopause.

[8] Mok S.K., Chen W.F., Lai W.P., Leung P.C., Wang X.L., Yao X.S., Wong M.S. (2010). Icariin protects against bone loss induced by oestrogen deficiency and activates oestrogen receptor-dependent osteoblastic functions in UMR 106 cells. Br. J. Pharmacol..

[9] Zhang Y., Li Q., Wan H.Y., Helferich W.G., Wong M.S. (2009). Genistein and a soy extract differentially affect three-dimensional bone parameters and bone-specific gene expression in ovariectomized mice. J. Nutr..

[10] Yamaguchi M. (2002). Isoflavone and Bone Metabolism: Its Cellular Mechanism and Preventive Role in Bone Loss. J. Health Sci..

[11] Chen X.W., Garner S.C., Anderson J.J. (2002). Isoflavones regulate interleukin-6 and osteoprotegerin synthesis during osteoblast cell differentiation via an estrogen-receptor-dependent pathway. Biochem. Biophys. Res. Commun..

[12] Windahl S.H., Lagerquist M.K., Andersson N., Jochems C., Kallkof A., Hakansson C., Inzunza J., Gustafsson J.A., Saag P.T., Calsten H. (2007). Identification of target cells for the genomic effects of estrogens in bone. Endocrinology.

[13] Clarkson T.B., Utian W.H., Barnes S., Gold E.B., Basaria S.S., Aso T., Kronenberg F., Frankenfeld C.L., Cline J.M., Landgren B.M. (2011). The role of soy isoflavones in menopausal health: Report of The North American Menopause Society/Wulf H. Utian Translational Science Symposium in Chicago, IL (October 2010). Menopause.

[14] Chen C.H., Ho M.L., Chang J.K., Hung S.H., Wang G.J. (2005). Green tea catechin enhances osteogenesis in a bone marrow mesenchymal stem cell line. Osteoporos. Int..

[15] Vali B., Rao L.G., El-Sohemy A. (2007). Epigallocatechin-3-gallate increases the formation of mineralized bone nodules by human osteoblast-like cells. J. Nutr. Biochem..

[16] Choi E.M., Hwang J.K. (2003). Effects of (+)-catechin on the function of osteoblastic cells. Biol. Pharm. Bull..

[17] Oka Y., Iwai S., Amano H., Irie Y., Yatomi K., Ryu K., Yamada S., Inagaki K., Oguchi K. (2012). Tea polyphenols inhibit rat osteoclast formation and differentiation. J. Pharmacol. Sci..

[18] Shen C.L., Yeh J.K., Cao J.J., Wang J.S. (2009). Green tea and bone metabolism. Nutr. Res..

[19] Sang S., Lambert J.D., Yang C.S. (2006). Bioavailability and stability issues in understanding the cancer preventive effects of tea polyphenols. J. Sci. Food Agric..

[20] Min K.R., Hwang B.Y., Lim H.S., Kang B.S., Oh G.J., Lee J., Kang S.H., Lee K.S., Ro J.S., Kim Y. (1999). (−)-*Epi*afzelechin: Cyclooxygenase-1 inhibitor and anti-inflammatory agent from aerial parts of Celastrus orbiculatus. Planta. Med..

[21] Kpegba K., Agbonon A., Petrovic A.G., Amouzou E., Gbeassor M., Proni G., Nesnas N. (2011). *Epi*afzelechin from the root bark of *Cassia sieberiana*: Detection by DART mass spectrometry, spectroscopic characterization, and antioxidant properties. J. Nat. Prod..

[22] Wu X.A., Zhao Y.M. (2005). Isolation and indentification of chemical compounds from *Drynaria fortunei*. China J. Chin. Mater. Med..

[23] Zeng X., Tian J., Cai K., Wu X., Wang Y., Zheng Y., Su Y., Cui L. (2014). Promoting osteoblast differentiation by the flavanes from Huangshan Maofeng tea is linked to a reduction of oxidative stress. Phytomedicine.

[24] Law M.C., Wong K.C., Pang W.Y., Wong M.S., Chan T.H. (2012). Chemical synthesis and biological study of 4 beta-carboxymethyl-*epi*afzelechin acid, an osteoprotective compound from the rhizomes of *Drynaria fortunei*. Medchemcomm.

[25] Wong K.C., Law M.C., Wong M.S., Chan T.H. (2014). Development of a UPLC-MS/MS bioanalytical method for the pharmacokinetic study of (−)-*epi*afzelechin, a flavan-3-ol with osteoprotective activity, in C57BL/6J mice. J. Chromatogr. B Analyt. Technol. Biomed. Life Sci..

[26] Wan S.B., Chan T.H. (2004). Enantioselective synthesis of afzelechin and *epi*afzelechin. Tetrahedron.

[27] Wang D., Christensen K., Chawla K., Xiao G., Krebsbach P.H., Franceschi R.T. (1999). Isolation and characterization of MC3T3-E1 preosteoblast subclones with distinct in vitro and in vivo differentiation/mineralization potential. J. Bone Miner. Res..

[28] Karsenty G. (2000). Role of Cbfa1 in osteoblast differentiation and function. Semin. Cell Dev. Biol..

[29] Cuetara B.L., Crotti T.N., O’Donoghue A.J., McHugh K.P. (2006). Cloning and characterization of osteoclast precursors from the RAW264.7 cell line. In Vitro Cell Dev. Biol. Anim..

[30] Khosla S. (2001). Minireview: The OPG/RANKL/RANK system. Endocrinology.

[31] Quarles L.D., Yohay D.A., Lever L.W., Caton R., Wenstrup R.J. (1992). Distinct proliferative and differentiated stages of murine MC3T3-E1 cells in culture: An in vitro model of osteoblast development. J. Bone Miner. Res..

[32] Zuo C., Huang Y., Bajis R., Sahih M., Li Y.P., Dai K., Zhang X. (2012). Osteoblastogenesis regulation signals in bone remodeling. Osteoporos. Int..

[33] Merriman H.L., Vanwijnen A.J., Hiebert S., Bidwell J.P., Fey E., Lian J., Stein J., Stein G.S. (1995). The Tissue-Specific Nuclear Matrix Protein, Nmp-2, Is a Member of the Aml/Cbf/Pebp2/Runt Domain Transcription Factor Family—Interactions with the Osteocalcin Gene Promoter. Biochemistry.

[34] Ducy P., Zhang R., Geoffroy V., Ridall A.L., Karsenty G. (1997). Osf2/Cbfa1: A transcriptional activator of osteoblast differentiation. Cell.

[35] Zhang Y., Li Q., Wan H.Y., Xiao H.H., Lai W.P., Yao X.S., Wong M.S. (2011). Study of the mechanisms by which Sambucus williamsii HANCE extract exert protective effects against ovariectomy-induced osteoporosis in vivo. Osteoporos. Int..

[36] Song D., Gan M., Zou J., Zhu X., Shi Q., Zhao H., Luo Z., Zhang W., Li S., Niu J. (2014). Effect of (−)-*epi*gallocatechin-3-gallate in preventing bone loss in ovariectomized rats and possible mechanisms. Int. J. Clin. Exp. Med..

[37] Peng Y., Yu B., Liu F. (2016). Epigallocatechin-3-gallate promotes osteoblastic activity in human osteoblast-like cells. Trop. J. Pharm. Res..

[38] Kamon M., Zhao R., Sakamoto K. (2009). Green tea polyphenol (−)-*epi*gallocatechin gallate suppressed the differentiation of murine osteoblastic MC3T3-E1 cells. Cell Biol. Int..

[39] Hammes S.R., Levin E.R. (2007). Extranuclear steroid receptors: Nature and actions. Endocr. Rev..

[40] Kousteni S., Han L., Chen J.R., Almeida M., Plotkin L.I., Bellido T., Manolagas S.C. (2003). Kinase-mediated regulation of common transcription factors accounts for the bone-protective effects of sex steroids. J. Clin. Invest..

[41] Almeida M., Han L., O’Brien C.A., Kousteni S., Manolagas S.C. (2006). Classical genotropic versus kinase-initiated regulation of gene transcription by the estrogen receptor alpha. Endocrinology.

[42] Lee M.J., Maliakal P., Chen L., Meng X., Bondoc F.Y., Prabhu S., Lambert G., Mohr S., Yang C.S. (2002). Pharmacokinetics of tea catechins after ingestion of green tea and (−)-*epi*gallocatechin-3-gallate by humans: Formation of different metabolites and individual variability. Cancer Epidemiol. Biomark. Prev..

[43] Chen L., Lee M.J., Li H., Yang C.S. (1997). Absorption, distribution, elimination of tea polyphenols in rats. Drug Metab. Dispos..

